# Plasma Adipokines in Patients Resuscitated from Cardiac Arrest: Difference of Visfatin between Survivors and Nonsurvivors

**DOI:** 10.1155/2020/9608276

**Published:** 2020-01-14

**Authors:** Yuan-Zhuo Chen, Shu-Qin Zhou, Yan-Qing Chen, Hu Peng, Yu-Gang Zhuang

**Affiliations:** Department of Emergency Medicine and Critical Care Medicine, Shanghai Tenth People's Hospital, School of Medicine, Tongji University, Shanghai, China

## Abstract

**Background:**

Adipokines are a group of cytokines or peptides secreted by adipose tissue to exert numerous biological functions. In the present study, we measured the plasma levels of four adipokines (adiponectin, leptin, fatty acid-binding protein 4 (FABP4), and visfatin) in cardiac arrest patients following return of spontaneous circulation (ROSC).

**Methods:**

Totally, 21 patients who experienced cardiac arrest and successful ROSC with expected survival of at least 48 hours (from January 2016 to December 2017) were consecutively enrolled into this prospective observational clinical study. Of the 21 enrolled patients, ten survived, and other eleven died between 2 days and 6 months post ROSC. Venous blood was drawn at three time points: baseline (<1 hour post ROSC), 2 days post ROSC, and 7 days post ROSC. Plasma concentrations of adiponectin, leptin, FABP4, and visfatin were determined using commercial enzyme-linked immunosorbent assays.

**Results:**

The plasma visfatin levels at 2 or 7 days post ROSC increased significantly compared with the baseline (*P* < 0.01), while plasma levels of adiponectin, leptin, and FABP4 did not change. Moreover, plasma visfatin levels in survivors at 2 or 7 days post ROSC were higher than those in nonsurvivors (*P* < 0.01). Plasma visfatin levels at 2 or 7 days post ROSC were negatively correlated with Acute Physiology and Chronic Health Evaluation (APACHE) II score and time to ROSC. Moreover, receiver operating characteristic curve analysis showed that the plasma visfatin levels at 2 or 7 days post ROSC were good predictors for survival of the patients.

**Conclusion:**

Elevated plasma visfatin levels may be a marker for better outcome of cardiac arrest patients post ROSC.

## 1. Introduction

Cardiac arrest is a major public health problem with substantial mortality and morbidity and affects more than one million people worldwide [1]. Although the survival rates for cardiac arrest vary widely among locations, it has not increased in parallel with the improvements in cardiopulmonary resuscitation (CPR) during recent years [2]. The postcardiac arrest syndrome (PCAS) is a period of critical entity following return of spontaneous circulation (ROSC) from cardiac arrest. Targeted temperature management by inducing hypothermia after ROSC has been thought to be one of the most promising therapies for patients with cardiac arrest [3]; however, it was mired in controversy since several recent findings indicated that hypothermia treatment was associated with a lower likelihood of survival to hospital discharge and a lower likelihood of favorable neurological survival [4]. Thus, assessing the prognosis of cardiac arrest patients with ROSC is rather important to identify and treat patients likely to have favorable neurological outcomes.

Adipokines is a group of cytokines or peptides secreted by adipose tissue, which lies at the central of obesity and adipose tissue dysfunction was proposed as a central mechanism connecting obesity with cardiovascular diseases. The obesity-related metabolic dysfunction may cause complicated influence on adipose biology and structure and thus affects the adipokine secretion and pattern. It should be noted that although adipokines were secreted from adipose tissue, the obesity/fat mass is not positively related to the secretion of adipokines. The circulating levels of adiponectin are reduced in patients with obesity [5], whereas another adipokine leptin levels in the blood are significantly increased in obesity patients [6]. Increased plasma visfatin concentration was found in morbidly obese subjects [7]. The dysregulated adipokines may be cause by the biological malfunction in adipose tissue such as inflammation. Moreover, it is well established that malfunction of adipokines provoked contributes to various cardiovascular diseases including vascular inflammation, hypertension, endothelial dysfunction, atherosclerosis, thrombosis/hemostasis, and vascular remodeling [8]. For example, adiponectin level is increased in left ventricle tissue in the rat model of postmyocardial infarction heart failure [9] and administration of adiponectin protected against the development of systolic dysfunction following myocardial infarction [10]. However, there is no study that assesses the change of adipokine in patients with cardiac arrest yet.

We speculated that the adipokines may be involved in neurological outcome and survival of cardiac arrest patients post ROSC. To this end, we measured the plasma levels of four adipokines (adiponectin, leptin, fatty acid-binding protein 4 (FABP4), and visfatin) in 21 cardiac arrest patients at the admission and 2 or 7 days post ROSC. Our results showed that plasma visfatin level was higher in the survivors than that in nonsurvivors. Moreover, the plasma visfatin levels at 2 or 7 days post ROSC were positively associated with the mortality and correlated with neurological outcome and time to ROSC.

## 2. Methods and Materials

### 2.1. Patients and Ethics Approval

This is a prospective observational study including 21 adult patients (age at least 18 years) admitted to the ICU of Department of Emergency Medicine at Shanghai Tenth People's Hospital from January 2016 to December 2017 following in- or out-of-hospital cardiac arrest event with subsequent CPR and ROSC. The inclusion criteria include (1) successful resuscitation after CPR; (2) admitted to ICU within 1 hour post ROSC; (3) age ≥ 18 and life expectancy >2 days; (4) written informed consent can be obtained. The exclusion criteria include (1) pregnant women; (2) drowning/hanging; (3) neuroendocrine tumor; (4) traumatic brain injury; (5) severe infection such as sepsis; (6) malignant. Of the 21 enrolled patients, ten survived, and other eleven died between 2 days and 6 months post ROSC.

The study was approved by the institutional review board of Shanghai Tenth People's Hospital, Tongji University, and registered with ClinicalTrial.gov on ClinicalTrial.gov (NCT02297776). This study was conducted in compliance with the 1964 Helsinki declaration and its later amendments. Written informed consents were obtained from the patient's next of kin at the return of spontaneous circulation (ROSC). If the patient regained consciousness, another written consent was obtained from the survivor.

### 2.2. Data Collection and Blood Sampling

All the CA patients were treated according to the standard intensive care protocol adopted locally. The Acute Physiology and Chronic Health Evaluation (APACHE) II score was calculated at the day of ICU enrolment as described previously [11]. Demographic information and other data including age, gender, past medical history, location of CA (in-hospital vs. out-of-hospital), and the cause of the events that led to cardiac arrest were collected. The time to ROSC was also recorded. Venous blood was drawn with a commercial EDTA tube (BD Medical Systems) at three time points: 1 hour, 2 days, and 7 days post ROSC. The blood samples were centrifuged immediately to remove cells and frozen (−80°C) after for further analysis. The basic blood parameters were measured by automated biochemical analyzer (HITACHI 7180, Japan).

### 2.3. Measurement of Adipokines

Plasma adipokine levels were analyzed twice for each sample using commercial enzyme-linked immunosorbent assays (ELISA) as described previously [12, 13]. The adiponectin ELISA assay (catalogue: DRP300, R&D Systems) detection limit was 0.891 ng/ml, and the assay range was 3.9-250 pg/ml. The resistin assay (catalogue: DRSN00, R&D Systems) detection limit was 0.055 ng/ml, and the assay range was 0.2-10 ng/ml. The FABP4 assay (catalogue: DFBP40, R&D Systems) detection limit was 14.2 ng/ml, and the assay range was 62.5-4,000 ng/ml. The visfatin assay (catalogue: K4907, BioVision, Inc., Minneapolis, MN) detection limit was 1.65 pg/ml, and the assay range was 6-400 pg/ml. All ELISA experiments were conducted according to the manufacturer's instructions as described previously [14–16].

### 2.4. Statistical Analysis

Firstly, to explore the fluctuation of adipokine levels after ROSC in all patients, we tested differences of adiponectin, resistin, FABP4, and visfatin among the three time points (1 hour, 2 days, and 7 days post ROSC). The adipokine levels are continuous variables and presented as the mean ± SEM. The Kruskal-Wallis test was used to test differences among three time points. Secondly, we divided the study subjects into two groups according the major outcome: whether short-term survival was achieved in the PCAS patients. Short-term survival was defined as survival for a minimum of 48 h after ROSC. We compared the differences of adipokine levels and other blood parameters between survivors or nonsurvivors at three time points. The Mann–Whitney *U* test was used to test differences between two groups [17]. Categorical variables (e.g., gender and comorbidities) are expressed as percentages. Frequencies were analyzed using either the chi-square test. Thirdly, to evaluate the accuracy of plasma adiponectin, resistin, FABP4, and visfatin levels to predict mortality, receiver operating characteristic (ROC) curves with the corresponding area under the curve (AUC) analyses were used. At last, the relations between adipokine levels and APACHE II score or time to ROSC were analyzed using the Pearson linear test. Statistical analyses were done using GraphPad Prism (GraphPad Prism Software, Inc., La Jolla, CA, USA). The threshold of significance was set at *P* < 0.05 [18, 19].

## 3. Results

### 3.1. Plasma Visfatin Level Shows an Increasing Trend Post ROSC in CA Patients

**Figure 1:**
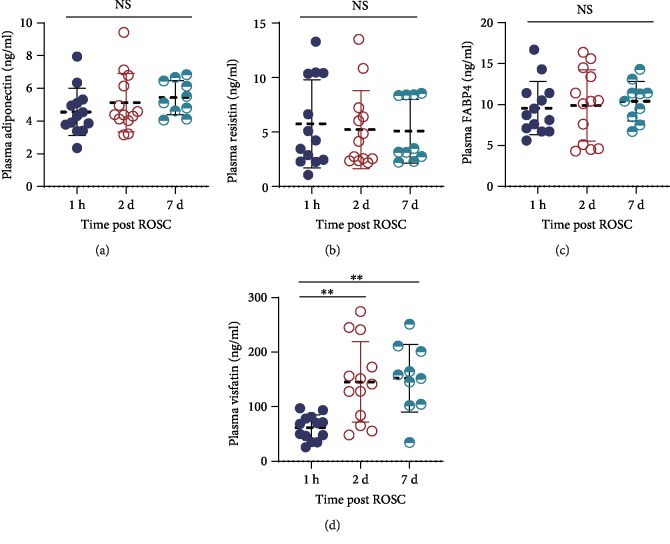
Violin plot shows the plasma levels of adiponectin (a), resistin (b), FABP4 (c), and visfatin (d) in PCAS patients at three time points (1 hour, 2 days, and 7 days post ROSC). The thick dotted line in violin plot indicates the median, whereas the two solid thin lines indicate the upper and lower quartiles. NS: no significance.

Table 1 shows the basic characteristics of the 21 PCAS patients. The time course changes (1 hour, 2 days, and 7 days post ROSC) of adiponectin, resistin, FABP4, and visfatin plasma levels in the 21 PCAS patients were studied. We did not observe any significant alterations in the plasma levels of adiponectin (Figure 1(a)), resistin (Figure 1(b)), and FABP4 (Figure 1(c)). However, we found the plasma visfatin levels at 2 and 7 days post ROSC were significantly higher than that at 1 hour post ROSC (*P* < 0.01, Figure 1(d)).

### 3.2. Plasma Visfatin Level Elevates in Survivors but Not in Nonsurvivors

**Figure 2:**
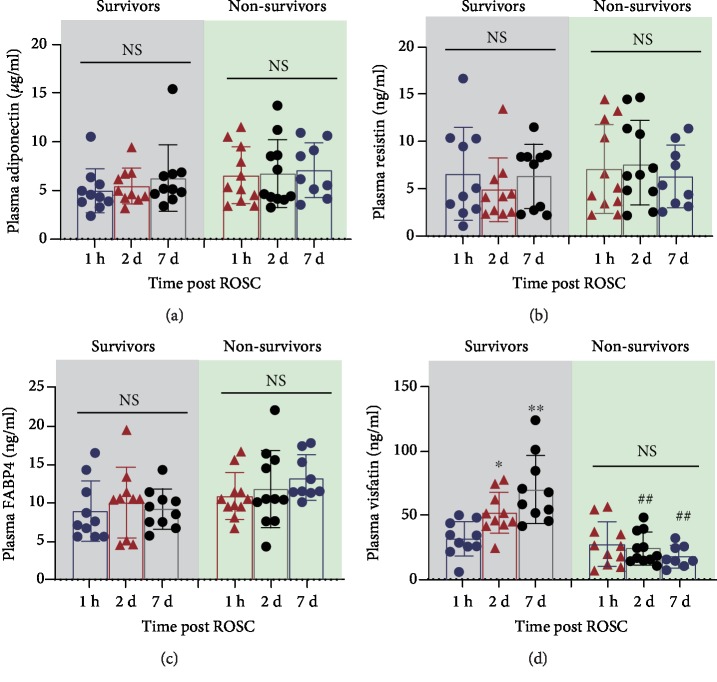
Scatter plot shows the plasma levels of adiponectin (a), resistin (b), FABP4 (c), and visfatin (d) in survivors and nonsurvivors of PCAS patients at three time points (1 hour, 2 days, and 7 days post ROSC). ^∗∗^*P* < 0.05, ^∗^*P* < 0.01 vs. 1 hour post ROSC. ^##^*P* < 0.01 nonsurvivors vs. survivors. NS: no significance.

Next, we grouped the 21 PCAS patients to the survivors (*n* = 10) and nonsurvivors (*n* = 11) for further analyses. There was no any significant difference of plasma adiponectin (Figure 2(a)), resistin (Figure 2(b)), and FABP4 (Figure 2(c)) among the three time points (1 hour, 2 days, and 7 days post ROSC) in survivors and nonsurvivors, respectively. In addition, the plasma levels of adiponectin (Figure 2(a)), resistin (Figure 2(b)), and FABP4 (Figure 2(c)) at three time points (1 hour, 2 days, and 7 days post ROSC) did not differ between the survivors and nonsurvivors. Interestingly, the plasma visfatin levels at 2 days (*P* < 0.01) and 7 days (*P* < 0.05) post ROSC were significantly higher than those at 1 hour post ROSC in the survivors but not in nonsurvivors (Figure 2(d)). Moreover, there were significant differences in the plasma visfatin levels at 2 and 7 days post ROSC between the survivors and nonsurvivors (Figure 2(d)). These results indicate that plasma visfatin level elevates in survivors but not in nonsurvivors.

### 3.3. Post-ROSC Plasma Visfatin Levels Are Negatively Associated with APACHE II Score in PCAS Patients

**Figure 3:**
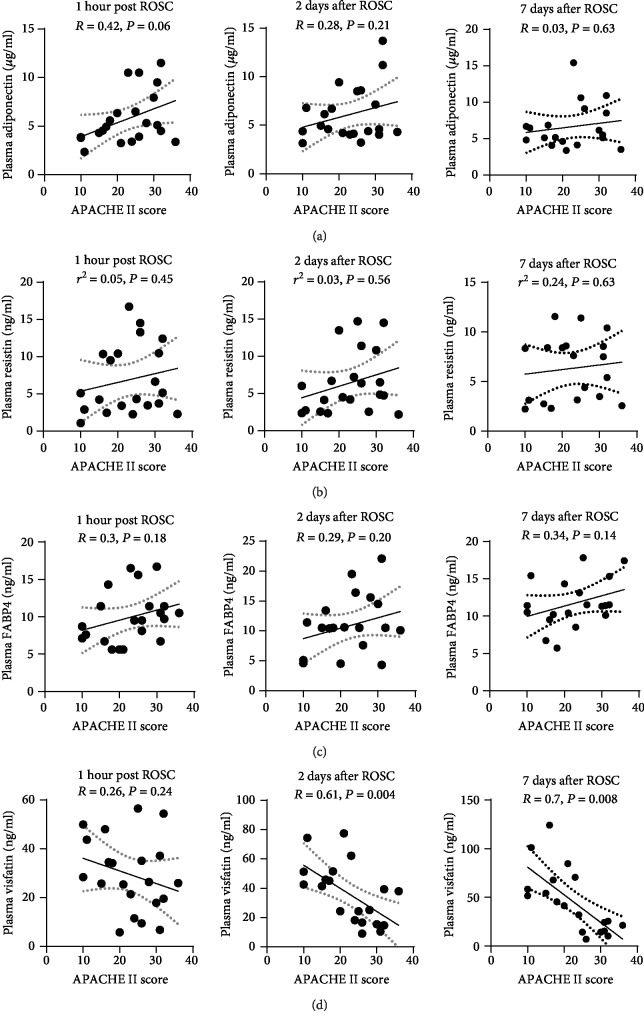
Association between plasma levels of adipokines (adiponectin, resistin, FABP4, and visfatin) and APACHE II score in PCAS patients. (a–d) Pearson linear regression analyses showed there were no associations between APACHE II score and plasma levels of diponectin (a), resistin (b), FABP4 (c), and visfatin (d) at three time points (1 hour, 2 days, and 7 days post ROSC). The dotted lines indicate 95% confidence interval (95% CI).

We investigated whether post-ROSC plasma adipokine levels might associate with the APACHE II score, a well-accepted mortality prediction tool for PCAS patients. Plasma levels of adiponectin, resistin, and FABP4 at three time points (1 hour, 2 days, and 7 days post ROSC) were not associated with APACHE II score (Figures 3(a)–3(c)). Although plasma level of visfatin at 1 hour post ROSC was not associated with APACHE II score, the plasma level of visfatin at 2 days and 7 days post ROSC was negatively associated with APACHE II score in PCAS patients (*P* = 0.004 and *P* = 0.008, respectively, Figure 3(d)).

### 3.4. Post-ROSC Plasma Visfatin Levels Are Negatively Associated with Time to ROSC in PCAS Patients

**Figure 4:**
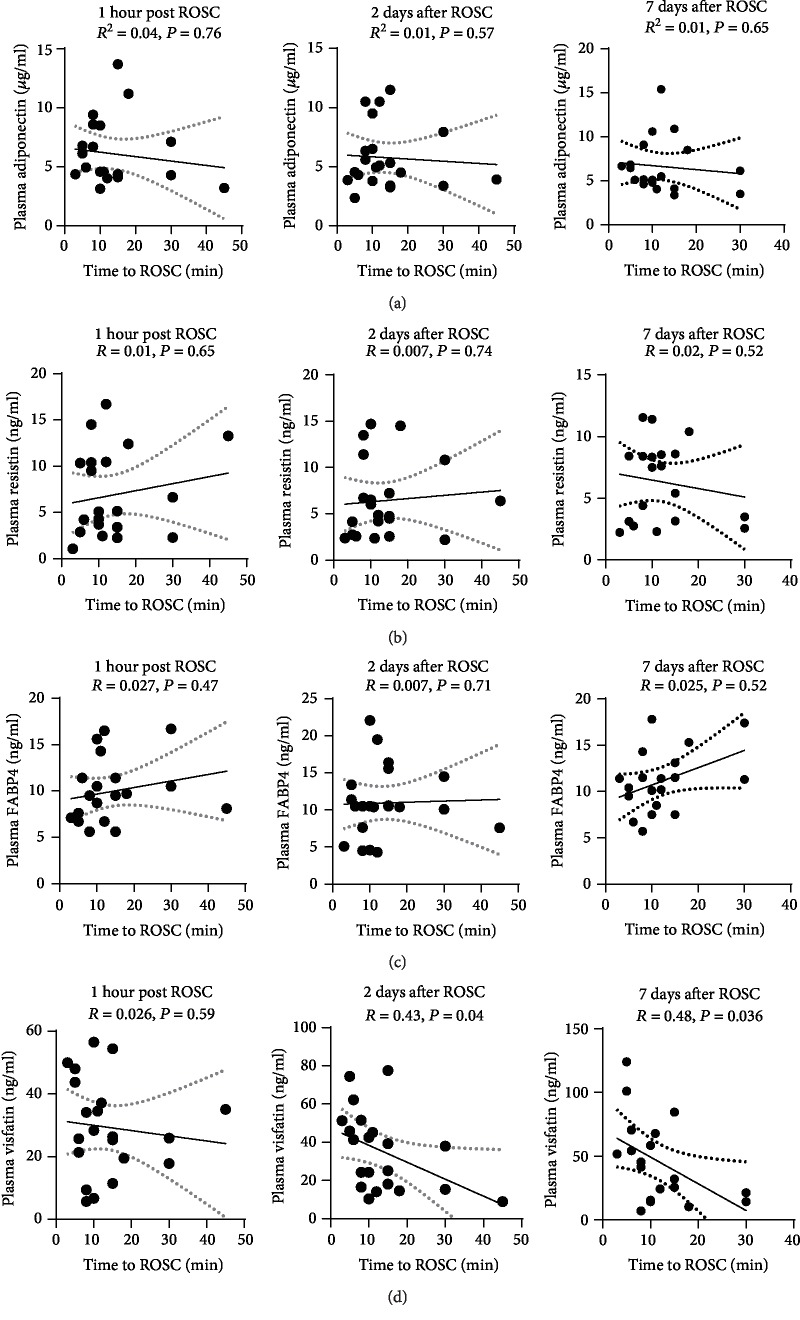
Association between plasma levels of adipokines (adiponectin, resistin, FABP4, and visfatin) and time to ROSC in PCAS patients. (a–d) Pearson linear regression analyses showed there were no associations between the time to ROSC and plasma levels of adiponectin (a), resistin (b), FABP4 (c), and visfatin (d) at three time points (1 hour, 2 days, and 7 days post ROSC). The dotted lines indicate 95% confidence interval (95% CI).

We also evaluated the associations between post-ROSC plasma levels of adipokines and time to ROSC. Plasma levels of adiponectin, resistin, and FABP4 at three time points (1 hour, 2 days, and 7 days post ROSC) were not associated with time to ROSC (Figures 4(a)–4(c)). Plasma level of visfatin at 1 hour post ROSC was still not associated with time to ROSC (Figure 4(d)). Plasma level of visfatin at 2 days and 7 days post ROSC was negatively associated with time to ROSC (*P* = 0.04 and *P* = 0.036, respectively, Figure 4(d)).

### 3.5. Post-ROSC Plasma Visfatin Level May Be a Predictor of Survival in PCAS Patients

**Figure 5:**
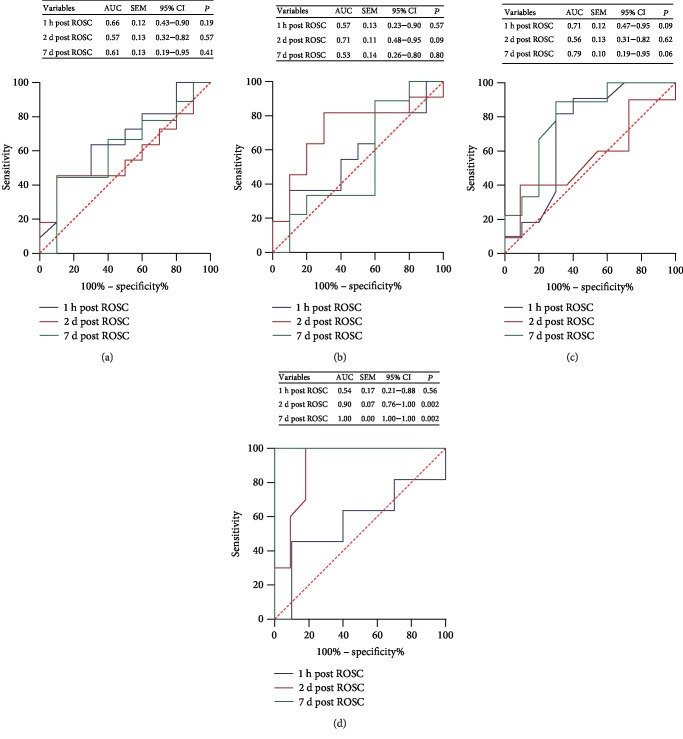
Plasma levels of visfatin may be a potential predictor of survival in PCAS patients. ROC curves for plasma levels of adiponectin (a), resistin (b), FABP4 (c), and visfatin (d) at three time points (1 hour, 2 days, and 7 days post ROSC) were assessed as possible predictors of survival in PCAS patients. The area under the curve (AUC), standard error of the mean (SEM), 95% confidence interval (95% CI), and *P* value were also illustrated.

At last, we analyzed the value of plasma levels of adiponectin, resistin, FABP4, and visfatin in predicting mortality. ROC analysis revealed that plasma levels of adiponectin and resistin at three time points (1 hour, 2 days, and 7 days post ROSC) were unable to predict the outcome of PCAS patients (Figures 5(a) and 5(b)). Similarly, plasma levels of FABP4 at 1 hour, 2 days, and 7 days post ROSC post ROSC were unable to predict the outcome (Figure 5(c)). However, the plasma levels of visfatin at both 2 days and 7 days post ROSC predicted well the outcome in PCAS patients (2 days post ROSC: AUC = 0.90, *P* = 0.002; 7 days post ROSC: AUC = 1.0, *P* = 0.002, Figure 5(d)).

## 4. Discussion

This is the first study to investigate the plasma concentrations of adipokines in PCAS patients and found plasma visfatin levels were enhanced post ROSC. Moreover, our results showed that plasma visfatin levels post ROSC only elevated in survivors but not in nonsurvivors. Finally, the ROC and linear analyses suggested that the plasma visfatin levels post ROSC were associated with APACHE II score and time to ROSC and thus may be a predictor of outcome of PCAS patients.

As a fast-growing problem that is reaching epidemic proportions worldwide, obesity contributes critically in the pathophysiology of cardiovascular diseases [20]. Adipose tissue lies at the central of obesity and adipose tissue dysfunction was proposed as a central mechanism connecting obesity with cardiovascular diseases. Accumulating knowledge on the biology and function of the adipose tissue has showed that the adipose tissue regulates cardiovascular health by secreting bioactive products such as adipokines and microvesicles, with a wide range of endocrine and paracrine effects on the cardiovascular system. Thus, the adipokines lie at the crossroad of nutrition, metabolism, and inflammation during progress of heart diseases especially coronary heart disease. However, there is an obesity paradox in cardiac arrest patients [21]. Jain et al. evaluated 21,237 adult in-hospital cardiac arrest patients and found that compared with overweight and obese patients, underweight, normal weight, and very obese had lower rates of survival to discharge [22]. Similarly, in a cohort study, Testori et al. reported that the overweight cardiac arrest patients with moderately elevated body mass index (BMI) may have a better neurological prognosis than lean patients (odds ratio 1.35; 95% confidence interval 1.02–1.79) [23]. Bunch et al. also observed that the normal- or low-weight patients with ventricular fibrillation in out-of-hospital cardiac arrest had a lower long-term survival compared with obese patients [24]. By contrary, several recent data indicate that obesity seemed to be associated with higher mortality [25]. The molecular mechanisms underlying this paradox are largely known.

Cardiac disease is still a worldwide health issue [26–29]. Notably, obesity contributes critically in the pathophysiology of cardiovascular diseases; however, there is an obesity paradox in cardiac arrest patients [21]. Jain et al. evaluated 21,237 adult in-hospital cardiac arrest patients and found that compared with overweight and obese patients, underweight, normal weight, and very obese had lower rates of survival to discharge [22]. Similarly, in a cohort study, Testori et al. reported that the overweight cardiac arrest patients with moderately elevated body mass index (BMI) may have a better neurological prognosis than lean patients (odds ratio 1.35; 95% confidence interval 1.02–1.79) [23]. Bunch et al. also observed that the normal- or low-weight patients with ventricular fibrillation in out-of-hospital cardiac arrest had a lower long-term survival compared with obese patients [24]. By contrary, several recent studies reported that obesity seemed to be associated with higher mortality [25]. The molecular mechanisms underlying this paradox are totally unknown. Accumulating knowledge on the biology and function of the adipose tissue has showed that the adipose tissue regulates cardiovascular health by secreting bioactive products such as adipokines and microvesicles, with a wide range of endocrine and paracrine effects on the cardiovascular system. Thus, the adipokines lie at the crossroad of nutrition, metabolism, and inflammation during progress of heart diseases. However, there is little information on serum adipokine concentrations in patients with cardiac arrest. Recently, Elmer et al. showed that serum leptin concentration did not differ between nonsurvivors and survivors [30]. This is the only investigation on the role of adipokines in cardiac arrest.

In this study, we selected four proinflammatory cytokines (adiponectin, resistin, FABP4, and visfatin), which have not been investigated in cardiac arrest yet, to explore their possible predict values in predicting outcome of patients post ROSC. We found plasma concentrations of adiponectin, resistin, and FABP4 did not differ between survivors and nonsurvivors. Only visfatin displayed a significant difference between the two groups. Moreover, visfatin correlated with neurological deficit score and time to ROSC and predicted the survival of patients well. Visfatin is a 54 kDa protein that was initially identified as a novel adipokine secreted by visceral fat with a putative insulin-mimetic property. Visfatin was identical to two previously described molecules named pre-B cell colony-enhancing factor (PBEF) and nicotinamide phosphoribosyltransferase (Nampt). Visfatin has been found to be an active player and potential drug target in cardiovascular diseases [31]. Two independent groups reported that plasma visfatin levels are significantly higher in patients with acute ST-elevation myocardial infarction [32, 33]. In patients with acute myocardial infarction, high visfatin level had good sensitivity and specificity (>70% and >75%, respectively) for predicting the incident [33]. Moreover, plasma visfatin level was associated with the occurrence of major adverse cardiovascular events in 185 patients with acute ST-elevation myocardial infarction [34]. Visfatin acts as an important inflammatory protein with enhanced expression in in macrophages of human unstable carotid and coronary atherosclerosis and associated with atherosclerotic plaque destabilization and thus acute coronary syndrome [35]. The expression of visfatin in circulating monocytes and neutrophils was also increased in male acute ST-segment elevation myocardial infarction patients [36]. These results indicated that visfatin might play a detrimental role in the development of atherosclerosis, atherosclerotic plaque destabilization, and ultimately myocardial infarction. Thus, it is expected that visfatin may be also a detrimental factor in cardiac arrest. Importantly, there is no previous experimental data on plasma visfatin concentration in patients with cardiac arrest. Unexpectedly, our results showed that plasma visfatin levels were higher in survivors compared with nonsurvivors and the high visfatin level in blood might be a good predictor of neurological outcome after ROSC.

There may be several explanations for our results. First, visfatin has cardioprotective effect due to its enzymatic activity in nicotinamide adenine dinucleotide (NAD^+^) biosynthesis. Lim et al. showed that intravenous administration of recombinant visfatin reduced the myocardial infarct size by ~50% in mouse model [37]. Hsu et al. also found visfatin significantly increased NAD^+^ and ATP concentrations in ischemic heart tissue, while cardiac-specific overexpression of visfatin reduced the infarction area and cardiac myocytes death in mouse ischemia/reperfusion model [38]. In agreement with these, treatment with visfatin enzymatic product nicotinamide mononucleotide in mice protected the heart from ischemia and reperfusion by decreasing acetylation of FoxO1 [39]. In this instance, the higher visfatin levels in blood of patients with cardiac arrest from ROSC may result in a cardioprotection in the ischemic heart tissue to some extent, which would affect the survival and neurological outcome of patients. Second, visfatin may exert a direct neuroprotection towards the ischemic brain during the time after ROSC. Since brain ischemia and neurologic deficit are the major determining factors of outcome in PCAS patients, the neuroprotective potential of visfatin may be involved in the beneficial action of visfatin in PCAS patients. Previously, a large number of studies have documented the potent neuroprotective action of visfatin in ischemic stroke [40]. In brain, visfatin is mainly expressed in neurons but not glia cells and the overexpression of visfatin upon ischemic stress in reduced ischemia-induced cerebral injuries in middle cerebral artery occlusion model through enhancing SIRT1-dependent AMPK activation [41]. This result was confirmed by several later investigations [42, 43]. The neuroprotection of visfatin may be attributed to many molecular mechanisms, including the boosting action on neuronal autophagy and neurogenesis [44]. Notably, plasma visfatin concentrations were elevated in patients with ischemic stroke [45]. Considering these, we proposed that the elevation of visfatin in blood may be an adaptive reaction in response to the acute ischemic stress during cardiac arrest. The patients who can motivated more visfatin into blood may have better resistance against ischemic stress. However, this speculation needs further experimental verification.

Our study has several limitations. First, the sample size is small, thus reducing the power of the study. As the present study is a single-center, observational study, only 21 patients were enrolled in the present study. We think the results would be more reliable if a greater size of sample will be applied. Second, we only obtained blood samples at time points. If blood is drawn at more time points, the changes of adipokines in cardiac arrest patients can be illustrated more accurately. Lastly, we only measured blood levels of four adipokines. Exploration on more adipokines in cardiac arrest patients may be an interesting topic to consider.

In summary, we for the first time to show that plasma visfatin may be a potential predictor of outcome of PCAS patients. Moreover, boosting systemic visfatin level or visfatin-regulated NAD^+^ pool may serve as a possible therapeutic target to enhance survival during post ROSC period in PCAS patients. Additional preclinical experiments and clinical trials with larger cohort sizes are warranted to test these concepts.

## Figures and Tables

**Table 1:** Patient basic characteristics.

	All patients (*N* = 21)
Age (years)	72.9
Sex, m/f	13/8
Witnessed by a bystander	15/21
Time to ROSC (min)	15.00
APACHE II score	21.06
CPC score	3.54
Out-of-hospital arrest	5/21
Ventricular defibrillation	7/21
Myocardial infarction	7/21
At presentation in ICU	
Cardiogenic shock	6/21
Postresuscitation sepsis	4/21
Length of ICU stay (days)	12.92
Length of hospital stay (days)	17.97

Data are expressed as average or absolute number where appropriate. ROSC: return of spontaneous circulation; ICU: intensive care unit; APACHE II: Acute Physiology and Chronic Health Evaluation II; CPC: cerebral performance category.

## Data Availability

The data used to support the findings of this study are included within the article.

## References

[1] Perkins G. D., Jacobs I. G., Nadkarni V. M. (2015). Cardiac arrest and cardiopulmonary resuscitation outcome reports: update of the Utstein Resuscitation Registry Templates for Out-of-Hospital Cardiac Arrest: a statement for healthcare professionals from a task force of the International Liaison Committee on Resuscitation (American Heart Association, European Resuscitation Council, Australian and New Zealand Council on Resuscitation, Heart and Stroke Foundation of Canada, InterAmerican Heart Foundation, Resuscitation Council of Southern Africa, Resuscitation Council of Asia); and the American Heart Association Emergency Cardiovascular Care Committee and the Council on Cardiopulmonary, Critical Care, Perioperative and Resuscitation. *Circulation*.

[2] Wang H. E., Kupas D. F. (2015). Outcomes after out-of-hospital cardiac arrest treated by basic vs advanced life support. *JAMA Internal Medicine*.

[3] Bernard S. A., Gray T. W., Buist M. D. (2002). Treatment of comatose survivors of out-of-hospital cardiac arrest with induced hypothermia. *The New England Journal of Medicine*.

[4] Chan P. S., Berg R. A., Tang Y., Curtis L. H., Spertus J. A., for the American Heart Association’s Get With the Guidelines–Resuscitation Investigators (2016). Association between therapeutic hypothermia and survival after in-hospital cardiac arrest. *Journal of the American Medical Association*.

[5] Ntzouvani A., Fragopoulou E., Panagiotakos D., Pitsavos C., Antonopoulou S. (2016). Reduced circulating adiponectin levels are associated with the metabolic syndrome independently of obesity, lipid indices and serum insulin levels: a cross-sectional study. *Lipids in Health and Disease*.

[6] Reinehr T., Kratzsch J., Kiess W., Andler W. (2005). Circulating soluble leptin receptor, leptin, and insulin resistance before and after weight loss in obese children. *International Journal of Obesity*.

[7] Haider D. G., Schindler K., Schaller G., Prager G., Wolzt M., Ludvik B. (2006). Increased plasma visfatin concentrations in morbidly obese subjects are reduced after gastric banding. *The Journal of Clinical Endocrinology and Metabolism*.

[8] Molica F., Morel S., Kwak B. R., Rohner-Jeanrenaud F., Steffens S. (2015). Adipokines at the crossroad between obesity and cardiovascular disease. *Thrombosis and Haemostasis*.

[9] Kalisz M., Baranowska B., Wolinska-Witort E. (2015). Total and high molecular weight adiponectin levels in the rat model of post-myocardial infarction heart failure. *Journal of Physiology and Pharmacology*.

[10] Shibata R., Izumiya Y., Sato K. (2007). Adiponectin protects against the development of systolic dysfunction following myocardial infarction. *Journal of Molecular and Cellular Cardiology*.

[11] Niskanen M., Kari A., Nikki P. (1991). Acute physiology and chronic health evaluation (APACHE II) and Glasgow coma scores as predictors of outcome from intensive care after cardiac arrest. *Critical Care Medicine*.

[12] Chen S., Wang Z., Xu B. (2015). The modulation of cardiac contractile function by the pharmacological and toxicological effects of Urocortin 2. *Toxicological Sciences*.

[13] Li C., Gao Y., Tian J., Xing Y., Zhu H., Shen J. (2012). Long-term oral Asperosaponin VI attenuates cardiac dysfunction, myocardial fibrosis in a rat model of chronic myocardial infarction. *Food and Chemical Toxicology*.

[14] Li D. J., Tong J., Zeng F. Y. (2019). Nicotinic ACh receptor *α*7 inhibits PDGF‐induced migration of vascular smooth muscle cells by activating mitochondrial deacetylase sirtuin 3. *British Journal of Pharmacology*.

[15] Li D. J., Liu J., Hua X. (2018). Nicotinic acetylcholine receptor *α*7 subunit improves energy homeostasis and inhibits inflammation in nonalcoholic fatty liver disease. *Metabolism*.

[16] Li D. J., Li Y. H., Yuan H. B., Qu L. F., Wang P. (2017). The novel exercise-induced hormone irisin protects against neuronal injury via activation of the Akt and ERK1/2 signaling pathways and contributes to the neuroprotection of physical exercise in cerebral ischemia. *Metabolism*.

[17] Siemian J. N., Wang K., Zhang Y., Li J. X. (2018). Mechanisms of imidazoline I_2_ receptor agonist-induced antinociception in rats: involvement of monoaminergic neurotransmission. *British Journal of Pharmacology*.

[18] Li D. J., Tong J., Li Y. H. (2019). Melatonin safeguards against fatty liver by antagonizing TRAFs‐mediated ASK1 deubiquitination and stabilization in a *β*‐arrestin‐1 dependent manner. *Journal of Pineal Research*.

[19] Rong B., Feng R., Liu C., Wu Q., Sun C. (2019). Reduced delivery of epididymal adipocyte-derived exosomal resistin is essential for melatonin ameliorating hepatic steatosis in mice. *Journal of Pineal Research*.

[20] Oikonomou E. K., Antoniades C. (2019). The role of adipose tissue in cardiovascular health and disease. *Nature Reviews Cardiology*.

[21] Chalkias A., Xanthos T. (2014). The obesity paradox in cardiac arrest patients. *International Journal of Cardiology*.

[22] Jain R., Nallamothu B. K., Chan P. S., for the American Heart Association National Registry of Cardiopulmonary Resuscitation (NRCPR) Investigators (2010). Body mass index and survival after in-hospital cardiac arrest. *Circulation: Cardiovascular Quality and Outcomes*.

[23] Testori C., Sterz F., Losert H. (2011). Cardiac arrest survivors with moderate elevated body mass index may have a better neurological outcome: a cohort study. *Resuscitation*.

[24] Bunch T. J., White R. D., Lopez-Jimenez F., Thomas R. J. (2008). Association of body weight with total mortality and with ICD shocks among survivors of ventricular fibrillation in out-of-hospital cardiac arrest. *Resuscitation*.

[25] Geri G., Savary G., Legriel S. (2016). Influence of body mass index on the prognosis of patients successfully resuscitated from out-of-hospital cardiac arrest treated by therapeutic hypothermia. *Resuscitation*.

[26] Prado N. J., Egan Beňová T., Diez E. R. (2019). Melatonin receptor activation protects against low potassium‐induced ventricular fibrillation by preserving action potentials and connexin‐43 topology in isolated rat hearts. *Journal of Pineal Research*.

[27] Ding M., Feng N., Tang D. (2018). Melatonin prevents Drp1-mediated mitochondrial fission in diabetic hearts through SIRT1-PGC1*α* pathway. *Journal of Pineal Research*.

[28] Lee F. T., Mountain A. J., Kelly M. P. (2005). Enhanced efficacy of radioimmunotherapy with 90Y-CHX-A''-DTPA-hu3S193 by inhibition of epidermal growth factor receptor (EGFR) signaling with EGFR tyrosine kinase inhibitor AG1478. *Clinical Cancer Research*.

[29] Lochner A., Marais E., Huisamen B. (2018). Melatonin and cardioprotection against ischaemia/reperfusion injury: What's new? A review. *Journal of Pineal Research*.

[30] Elmer J., Jeong K., Abebe K. Z. (2016). Serum neutrophil gelatinase-associated lipocalin predicts survival after resuscitation from cardiac arrest. *Critical Care Medicine*.

[31] Romacho T., Sanchez-Ferrer C. F., Peiro C. (2013). Visfatin/Nampt: an adipokine with cardiovascular impact. *Mediators of Inflammation*.

[32] Lu L. F., Wang C. P., Yu T. H. (2012). Interpretation of elevated plasma visfatin concentrations in patients with ST- elevation myocardial infarction. *Cytokine*.

[33] Mazaherioun M., Hosseinzadeh-Attar M. J., Janani L. (2012). Elevated serum visfatin levels in patients with acute myocardial infarction. *Archives of Iranian Medicine*.

[34] Hung W. C., Yu T. H., Hsu C. C. (2015). Plasma visfatin levels are associated with major adverse cardiovascular events in patients with acute ST-elevation myocardial infarction. *Clinical and Investigative Medicine*.

[35] Dahl T. B., Yndestad A., Skjelland M. (2007). Increased expression of visfatin in macrophages of human unstable carotid and coronary atherosclerosis: possible role in inflammation and plaque destabilization. *Circulation*.

[36] Chiu C. A., Yu T. H., Hung W. C. (2012). Increased expression of visfatin in monocytes and macrophages in male acute myocardial infarction patients. *Mediators of Inflammation*.

[37] Lim S. Y., Davidson S. M., Paramanathan A. J., Smith C. C. T., Yellon D. M., Hausenloy D. J. (2008). The novel adipocytokine visfatin exerts direct cardioprotective effects. *Journal of Cellular and Molecular Medicine*.

[38] Hsu C. P., Oka S., Shao D., Hariharan N., Sadoshima J. (2009). Nicotinamide phosphoribosyltransferase regulates cell survival through NAD+ synthesis in cardiac myocytes. *Circulation Research*.

[39] Yamamoto T., Byun J., Zhai P., Ikeda Y., Oka S., Sadoshima J. (2014). Nicotinamide mononucleotide, an intermediate of NAD+ synthesis, protects the heart from ischemia and reperfusion. *PLoS One*.

[40] Wang P., Miao C. Y. (2015). NAMPT as a therapeutic target against stroke. *Trends in Pharmacological Sciences*.

[41] Wang P., Xu T. Y., Guan Y. F. (2011). Nicotinamide phosphoribosyltransferase protects against ischemic stroke through SIRT1-dependent adenosine monophosphate-activated kinase pathway. *Annals of Neurology*.

[42] Erfani S., Khaksari M., Oryan S., Shamsaei N., Aboutaleb N., Nikbakht F. (2015). Nampt/PBEF/visfatin exerts neuroprotective effects against ischemia/reperfusion injury via modulation of Bax/Bcl-2 ratio and prevention of caspase-3 activation. *Journal of Molecular Neuroscience*.

[43] Wei C. C., Kong Y. Y., Hua X. (2017). NAD replenishment with nicotinamide mononucleotide protects blood-brain barrier integrity and attenuates delayed tissue plasminogen activator-induced haemorrhagic transformation after cerebral ischaemia. *British Journal of Pharmacology*.

[44] Zhao Y., Guan Y. F., Zhou X. M. (2015). Regenerative neurogenesis after ischemic stroke promoted by nicotinamide phosphoribosyltransferase-nicotinamide adenine dinucleotide cascade. *Stroke*.

[45] Lu L. F., Yang S. S., Wang C. P. (2009). Elevated visfatin/pre-B-cell colony-enhancing factor plasma concentration in ischemic stroke. *Journal of Stroke and Cerebrovascular Diseases*.

